# High-power-short-duration-radiofrequency-, cryoballoon-, and pulsed-field-ablation: a prospective clinical study

**DOI:** 10.1186/s12872-026-05641-y

**Published:** 2026-02-25

**Authors:** Daniel Robinson, Hanna Seiler, Stephan Valenta, Plamen Kochev, Jakob Eichhorn, Jonas Bodanowitz, Anton Khvan, Annegret Werner, Chrisia Arnold, Christian Ewertsen, Sebastian Feickert, Burkert Pieske, Hüseyin Ince, Jasmin Ortak

**Affiliations:** 1 Department of Cardiology, University of Rostock, Ernst-Heydemann-Strasse 6, Rostock, 18057 Germany; 2 Department of Cardiology, Vivantes Klinikum am Urban, Dieffenbachstrasse 1, Berlin, 10967 Germany

**Keywords:** Atrial fibrillation, Pulmonary vein isolation, Pulsed-field ablation, High-power-short-duration-radiofrequency ablation, Cryoballoon ablation

## Abstract

**Background:**

Pulmonary vein isolation aims to reduce the burden of atrial fibrillation leading to symptom alleviation and improvement in quality of life. The newer pulsed-field ablation offers a non-thermal alternative to radiofrequency- and cryoballoon- ablation. We present one of the first clinical, pilot prospective comparisons of these three procedures with respect to freedom from recurrence up to one year, procedure safety, and assessment of quality of life.

**Methods:**

Localization of the pulmonary veins during pulsed-field ablation and radiofrequency ablation was supported by electroanatomical mapping, whereas only fluoroscopy was used for cryoballoon ablation. The ablation techniques also differed in terms of energy delivery. Pulsed-field ablation and cryoballoon ablation used a single-shot technique, while radiofrequency ablation used high-power, short-duration energies (50 W/10 Seconds) with point-by-point lesions. The study included 36 patients, 15 of whom were treated with pulsed-field ablation, 10 with high-power, short-duration radiofrequency ablation and 11 with cryoballoon ablation.

**Results:**

Cryoballoon ablation showed the shortest ablation and catheter-dwell times. The fluoroscopy time and the dose area product were lowest for radiofrequency ablation. No recurrences of atrial fibrillation were recorded in the cryoballoon ablation group during the follow-up period, whereas 26.7% of participants in the pulsed-field ablation group and 30.0% in the radiofrequency ablation did. These differences, however, were not statistically significant. Quality of life showed a significant improvement in all three study groups.

**Conclusions:**

All three ablation procedures achieved the goal of reducing symptom burden with no statistically significant differences with respect to atrial fibrillation recurrence. Due to the great epidemiological significance of the disease, larger studies are needed to evaluate the success of the three ablation methods.

## Introduction

Atrial fibrillation is the most common cardiac arrhythmia and is often associated with considerable subjective stress for those affected [1, 2]. In addition to the individual reduction in quality of life [3], the disease poses a high risk of complications and comorbidities. In 1998, the pulmonary veins were discovered to be an important site for triggering atrial fibrillation. It was shown that focal electrical activity can both induce and maintain atrial fibrillation. It was also found that the foci respond to therapeutic intervention by means of ablation [4, 5]. Radiofrequency (RF) is a well-established ablation strategy in the management of atrial fibrillation and lesion characteristics differ depending on the level of RF-power used and its duration [6]. Many studies have shown that high-power short-duration (HPSD)-RF leads to shorter procedure times whilst maintaining good lesion durability compared to the standard low-power long-duration (LPLD)-RF [7–9]. Thermal-based catheter ablation has also expanded to include cryoballoon-technology with a faster, single-shot technique compared to the more time-consuming point-by-point RF-procedure. In the Fire and Ice study, which investigated the success and safety profile of the RF- and cryoballoon procedures, non-inferiority of cryoballoon ablation was demonstrated [10]. In addition to these thermal procedures, pulsed field ablation (PFA) has emerged as a new therapeutic approach with a non-thermal energy output combined with a single-shot technique [11]. The procedure uses electric fields to induce irreversible electroporation. One advantage of PFA is its tissue-specificity and surrounding structures, such as the esophagus, have higher threshold values for the onset of electroporation than cardiomyocytes. This allows the procedure to treat the targeted tissue area [11]. It is hoped that this newer ablation method will not only have an improved safety profile, but also higher success rates in terms of post-interventional recurrence rates.

The aim of this scientific paper is to compare the ablation methods pulsed field ablation (PFA), high-power, short-duration radiofrequency (HPSD-RF) ablation and cryoballoon ablation in atrial fibrillation. As a rhythm-preserving therapeutic approach, catheter ablation shows promising results and while there are already studies investigating the individual procedures, there is still a lack of information on the newer PFA method in comparison to radiofrequency ablation and cryoballoon ablation. The following study is the first to compare the effectiveness and the safety of PFA with HPSD-RF und cryoballoon ablation. In addition, the three procedures are compared with regard to the intra-individual change in quality of life before and after the intervention.

## Materials & methods

The present study is a clinical, pilot prospective study involving patients undergoing elective pulmonary vein isolation for symptomatic, paroxysmal atrial fibrillation at the University of Rostock between September 2021 and May 2023. Patients were assigned non-randomly to an ablation group (either PFA, HPSD-RF ablation or cryoballoon ablation). The study was approved by the local ethic commission of the medical department of the university of Rostock. All patients gave their informed consent. All data were anonymized. Clinical data included age, sex, height, weight, previous illnesses, medications, clinical symptoms, the duration of atrial fibrillation since first diagnosis, the CHA_2_DS_2_-VASc-Score, and blood pressure. Additionally, left atrial (LA) diameter, left ventricular systolic function (LV-EF) and the presence of any heart valvular pathology were assessed in a pre-interventional transesophageal echocardiography (TEE). A pre-interventional 12-lead electrocardiogram (ECG) recording was also made. Clinical symptoms and subjective assessment of quality of life were made using the Atrial Fibrillation Effect on Quality of life (AFEQT) and the EuroQol-5 Dimensionas-5-Levels (EQ-5D-5 L) questionnaires. Procedural Data included procedural duration, catheter duration in the left atrium, contrast agent volume, fluoroscopy time and the dose area product. All medication used during the procedure as well as any complications were documented. All patients were followed-up at 1 month, 3 months, 6 months and 12 months, each time with a 12-lead ECG as well as a 24 h-ECG-recording. Any health problems or events that occurred during the follow-up period were also documented. The quality of life questionnaires were also re-assessed at follow-ups 3, 6 and 12 months. In case of any unplanned clinical presentations, a 12-lead ECG was recorded to detect any recurrence of the atrial fibrillation.

### Inclusion and exclusion criteria

Patients with symptomatic, paroxysmal atrial fibrillation who had not undergone any previous pulmonary vein isolation were included in the study, regardless of any prior antiarrhythmic medication and who were > 18 years of age. Exclusion criteria were a large left atrium (LA-Diameter > 50 mm), a reduced systolic LV-Function (LV-EF < 45%), atrial fibrillation with a reversible cause, high-grade mitral valve regurgitation or stenosis, previous myocardial infarction, percutaneous coronary intervention (PCI) or heart surgery in the last 3 months prior to enrollment, congenital heart disease, heart failure with New York Heart Association (NYHA) III-IV, pregnancy, age < 18 years, a life expectancy of < 1 year or kidney-failure patients receiving dialysis.

### Study endpoints

The study endpoint was a recurrence of atrial fibrillation occurring more than 3 months after pulmonary vein isolation. All recurrences had to be documented in an ECG (12 -lead and/or 24 h-ECG) or as an atrial tachycardia for > 30 s.

### Pulmonary vein isolation

A TEE was performed to exclude intracardial thrombosis before every procedure. Patients were intravenously sedated using propofol, midazolam and fentanyl. Procedural monitoring included oxygen saturation levels, ECG and heart rate and blood pressure was monitored with use of an arterial line (usually inserted in the left radial artery). Patients were heparinized and regular, activated clotting times (Target: >300 s) were measured to ensure adequate anticoagulation during the procedure. Direct oral anticoagulants (DOAC) were paused on the day of the procedure. Patients receiving vitamin K antagonists such as Warfarin, underwent the procedure without any adjustment. Further medication with atropine, noradrenaline or pantoprazole was given as required. Patients with a known contrast-agent allergy received an allergy-prophylaxis (Prednisolon 100 mg i.v, Cimetidin 200 mg i.v. and Clemastinfurat 2 mg i.v.) before undergoing PFA or Cryoballoon ablation.

The right femoral vein was used as access for the intracardial catheters. Procedural duration began with insertion of the first needle into the femoral vein. A 10-pole-catheter (Inquiry, Abbott Laboratories, Chicago, USA) was introduced into the coronary sinus and a Josephson catheter (Abbott Laboratories, Chicago, USA) was used for His-documentation. All atrial transseptal punctures occurred under fluoroscopy and measurement of atrial pressure. A temperature sensor was placed in the esophagus for the thermal ablation therapies (SensiTherm, Abbot Laboratories, Chicago, USA). The CARTO^®^ mapping system (Biosense Webster, Johnson & Johnson, New Brunswick, USA) was used to construct the atrial-electroanatomical map for PFA- and HPSD-RF ablations.

The Farapulse^®^ system (Boston Scientific Corporation, Marlborough, USA) was used for PFA. Only one atrial-septal puncture was needed for PFA. Each pulmonary vein received at least 4 “basket” ablations and 4 “flower” ablations or more, until complete electrical isolation of the vein was achieved. Fluoroscopic control was additionally used to confirm the Farawave^®^ catheter position.

The Thermocool-Smarttouch™ System (SF- and ST-catheters and LASSO-Mapping-Catheter; Biosense Webster, Johnson & Johnson, New Brunswick, USA) was used for HPSD-RF ablation. Patients undergoing HPSD-RF underwent two atrial-septal punctures for introduction of the mapping and ablation catheters. The Agilis™NxT steerable introducer (Abbott Laboratories, Chicago, USA) was additionally used to help guide mapping and ablation. Radiofrequency ablation was performed using the High Power Short Duration (HSPD) technique with an energy application of 50 Watts for a duration of 10 s on a point-for-point basis and guided by the Ablation Index. These points followed a circumferential line around the pulmonary veins until electrical isolation was achieved.

The Artic Front Advance System™ (Medtronic, Dublin, Ireland) was used for cryoballoon ablation. Only one atrial-septal puncture was needed for cryoballoon ablation. An angiography of the left atrium and pulmonary veins was performed under fast stimulation of the right ventricle in each patient to assist in the placement of the cryoballoon. Each pulmonary vein was ablated under stimulation of the phrenic nerve with a temperature of almost − 60 °C and a duration of approximately 240 s.

Following each ablation treatment, electrical isolation was demonstrated for entrance- and exit-block in each pulmonary vein. The catheters were removed under fluoroscopic control. The inguinal punctures were closed with a “Z”-suture and a manual compression bandage applied as well as bedrest for 12 h. A post-procedural echocardiography was performed to rule out pericardial effusion. Discharge from hospital occurred the following day by an uncomplicated procedure and patients received full anticoagulation regardless of the CHA_2_DS_2_-VASc-Score for at least 12 weeks. Additionally, a proton-pump-inhibitor was given for 4–6 weeks following the ablation procedure.

The AFEQT questionnaire assessed the quality of life in patients with atrial fibrillation [12]. A change in its score of +/-5 is seen as clinically relevant [12]. The EQ-5D-5 L questionnaire assessed characteristics such as mobility, self-care, daily routines, pain, symptoms, fear and depression [13]. In each category, participants could choose between five statements and thus indicate the extent to which problems existed in the respective area. A value was formed from the dimensions, which was transformed into an index with the best value of 1 in accordance with the country-specific value set for Germany [14]. In addition, a percentage was used to assess personal health at the current time using a visual analog scale.

### Statistics

All data collected was analyzed using the software programs Excel (version 2018) and SPSS (IPM Statistics version 27). Nominal and ordinal variables were presented descriptively with frequencies, whereas metric distribution-free variables were presented using means and quartiles 1 and 3. Nominal variables were tested for differences using a cross-tabulation and the chi-square test. All metric variables were tested for normal distribution. This was done using a graphical assessment in the form of a histogram and a Q-Q diagram, as well as by means of an examination using the Shapiro-Wilk test. Due to the relatively small sample size, distribution-free statistical tests were preferred in individual cases. Independent metric and distribution-free variables, as well as ordinal variables, were tested for differences when comparing the three samples using the Kruskal-Wallis test. If only two samples were to be compared in an independent metric and distribution-free variable, the Mann-Whitney U test was used. The dependent, distribution-free variables were analyzed using the Friedman test. In the case of significant results between the groups or measurement times, a pairwise analysis was performed. A survival time analysis using the Kaplan-Meier curve was used to illustrate atrial fibrillation recurrence. The event rates of the groups were then checked using the log-rank test. A significance level of 0.05 was set for the decision to accept or reject the null hypothesis in statistical tests. If the test revealed a difference between the three groups or measurement times, this was investigated further with the help of a pairwise comparison. In order to avoid alpha-error accumulation, the significance level was adjusted according to the Bonferroni correction.

## Results

A total of 36 patients were enrolled in the study. Fifteen received PFA, ten received HPSD-RF and eleven received cryoballoon ablation. The baseline characteristics of the study participants are shown in Table 1 and were comparable in each of the ablation-strategy groups with no statistically significant differences. Patients undergoing HPSD-RF tended to report a longer duration of atrial fibrillation.

**Table 1:** Baseline Characteristics

	Pulsed-Field Ablation	Radiofrequency Ablation	Cryoballoon Ablation	*p*-Value
Subjects (*n*)	15	10	11	
Age, median (years) (Q1-Q3)	64.68 (55.62–73.43)	69.54 (59.80-74.88)	68.68 (62.09–72.60)	0.819
Male Sex (*n* (%))	6 (40.0)	4 (40.0)	3 (27.3)	0.765
BMI, median (kg/m²) (Q1-Q3)	29.30 (24.20–31.00)	27.55 (26.23–31.68)	31.90 (28.90–34.50)	0.099
Systolic Blood Pressure, median (mmHg) (Q1-Q3)	134 (125–145)	140 (125.25–142.50)	148 (138–150)	0.346
Diastolic Blood Pressure, median (mmHg) (Q1-Q3)	80 (77–80)	80 (70,0–88,75)	79 (72–83)	0.754
Duration of atrial fibrillation, median (months) (Q1-Q3)	22 (10–94)	36 (13.25–73.25)	20 (14–37)	0.856
Median LV-EF in %: (Q1-Q3)	56,00 (55,00–59,00)	58,50 (54,75 − 63,50)	55,00 (55,00–62,00)	0,582
LA-Diameter, median (mm) (Q1-Q3)	39.00 (35.00–45.00)	39.50 (34.25-47.00)	40.00 (34.00–42.00)	0.875
CHA_2_DS_2_-VASc-Score, median (Q1-Q3)	3.00 (2.00–4.00)	2.50 (2.00-4.25)	3.00 (2.00–4.00)	0.674
*n* (%):0	1 (6.7)	0 (0.0)	0 (0.0)	
*n* (%):1	2 (13.3)	0 (0.0)	1 (9.1)	
*n* (%):2	4 (26.7)	5 (50.0)	2 (18.2)	
*n* (%):3	4 (26.7)	1 (10.0)	4 (36.4)	
*n* (%):4	2 (13.3)	2 (20.0)	3 (27.3)	
*n* (%):5	2 (13.3)	2 (20.0)	1 (9.1)	
NYHA-Status, median (Q1-Q3)	0.0 (0.0–0.0)	0.0(0.0-0.5)	0.0(0.0–1.0)	0.412
*n* (%):0	14 (93.3)	8 (80.0)	8 (72.7)	
*n* (%):I	0 (0.0)	0 (0.0)	2 (18.2)	
*n* (%):II	1 (6.7)	1 (10.0)	1 (9.1)	
*n* (%):III	0 (0.0)	1 (10.0)	0 (0.0)	
Comorbidities and cardiovascular risk factors (*n* (%))				
Hypertension	11 (73.3)	8 (80.0)	10 (90.9)	0.534
Previous Stroke or TIA	3 (20.0)	1 (10.0)	0 (0.0)	0.274
Diabetes mellitus Type 2	2 (13.3)	4 (40.0)	3 (27.3)	0.314
Hyperlipidemia	5 (33.3)	3 (30.0)	5 (45.5)	0.730
Coronary Artery Disease	3 (20.0)	3 (30.0)	1 (9.1)	0.480
Chronic Kidney Disease	0 (0.0)	1 (10.0)	1 (9.1)	0.467
Medications (*n* (%))				
DOAC	13 (86.7)	9 (90.0)	10 (90.9)	0.936
Vitamin-K-Antagonist	1(6.7)	0 (0.0)	0 (0.0)	0.487
Platelet Inhibitors	2 (13.3)	2 (20.0)	0 (0.0)	0.373
Antiarrhythmic Agents Class Ic	4 (26.7)	2 (20.0)	2 (18.2)	0.859
Antiarrhythmic Agents Class III	1 (6.7)	1 (10.0)	0 (0.0)	0.589
Betablocker	11 (73.3)	9 (90.0)	10 (90.9)	0.396

Table 2 shows the procedural data. The duration of the procedure describes the time from the start of the procedure through the inguinal puncture until the catheter is withdrawn from the body. The catheter dwell time is the time during which the catheter was in the left atrium. Both times were shortest in the cryoballoon ablation group with a mean procedure time of 1:19 h and a catheter dwell time of 1:01 h. There was a significant difference in the duration of ablation between the groups (*p*-value = 0.022). In the further pairwise analysis, no difference was detected between PFA and cryoballoon ablation (*p* = 0.560), or PFA and HPSD-RF (*p* = 0.289), but there was a significant difference between HPSD-RF and cryoballoon ablation (*p* = 0.018). The comparison of catheter dwell times also showed detectable differences in the statistical test (*p* = 0.006). In the pairwise analysis, there was no detectable difference between the PFA and cryoballoon ablation groups (*p* = 0.293), nor in the comparison of PFA with HPSD-RF (*p* = 0.216). There was a difference between the HPSD-RF and cryoballoon ablation groups (*p* = 0.004).

**Table 2:** Procedural Data

	Pulsed-Field Ablation	Radiofrequency Ablation	Cryoballon Ablation	*p*-Value
Procedure Duration, median (hours: minutes: seconds)(Q1-Q3)	1:24:00 (1:16:00–1:59:00)	1:42:00 (1:31:30 − 1:56:00)	1:19:00 (1:13:00–1:26:00)	0.022
Catheter Dwell-Time, median (hours: minutes: seconds) (Q1-Q3)	1:06:00 (1:02:10 − 1:47:33)	1:30:00 (1:23:45 − 1:32:45)	1:01:00 (0:48:00–1:07:00)	0.006
Fluoroscopy time, median (minutes: seconds) (Q1-Q3)	35:16 (28:11–37:51)	07:25 (06:47 − 08:80)	15:40 (14:46 − 16:04)	< 0.001
Dose Area Product, median (µGym²) (Q1-Q3)	1507.10 (961.35-2021.16)	340.45 (251.83-516.05)	1115.80 (941.80-1376.10)	< 0.001

**Fig. 1:**
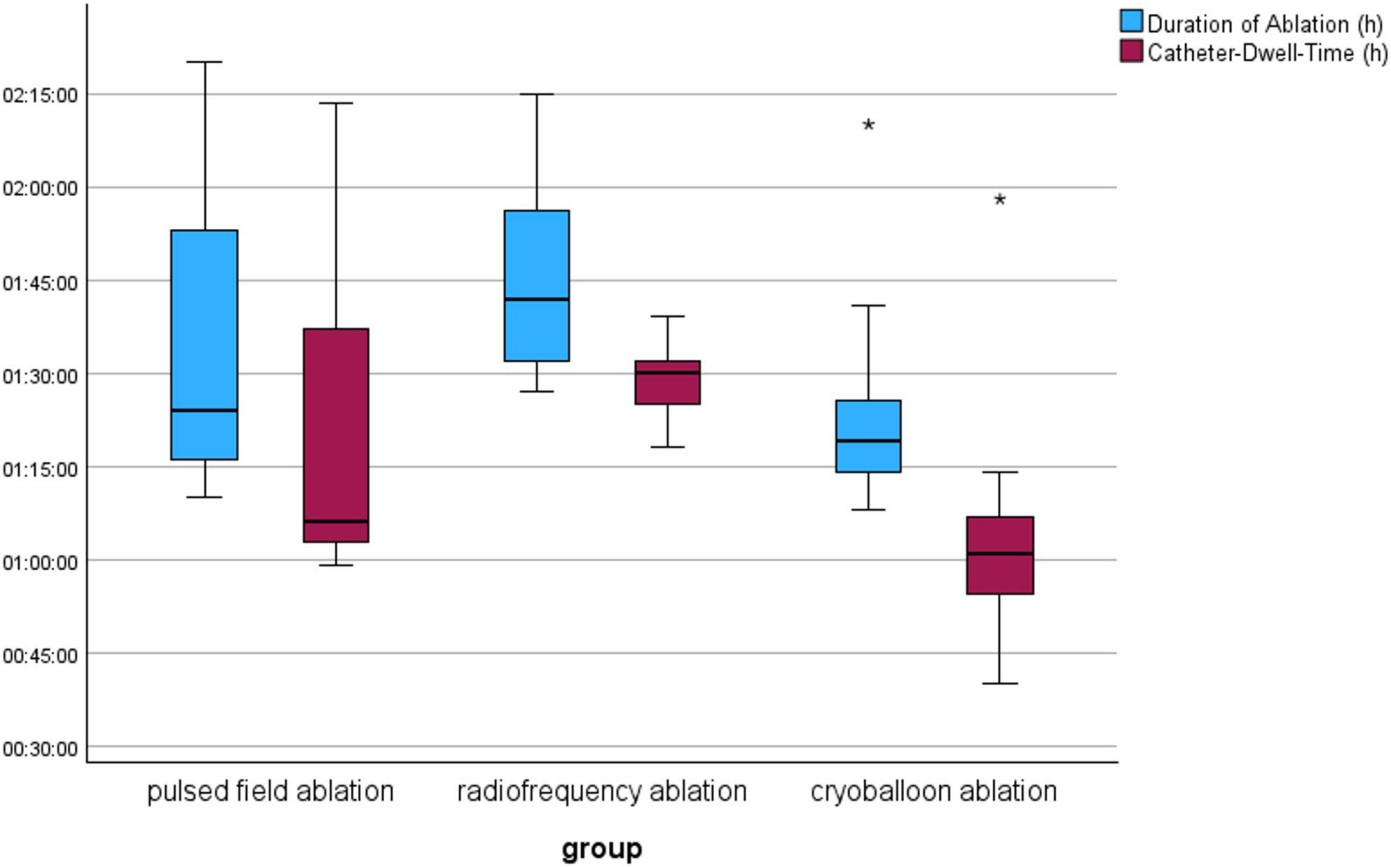
Duration of Ablation Procedure (*p* = 0.022) and Catheter-Dwell-Time (*p* = 0.006), Legend: * denotes an outlier due to procedure difficulty (see text for explanation)

**Fig. 2:**
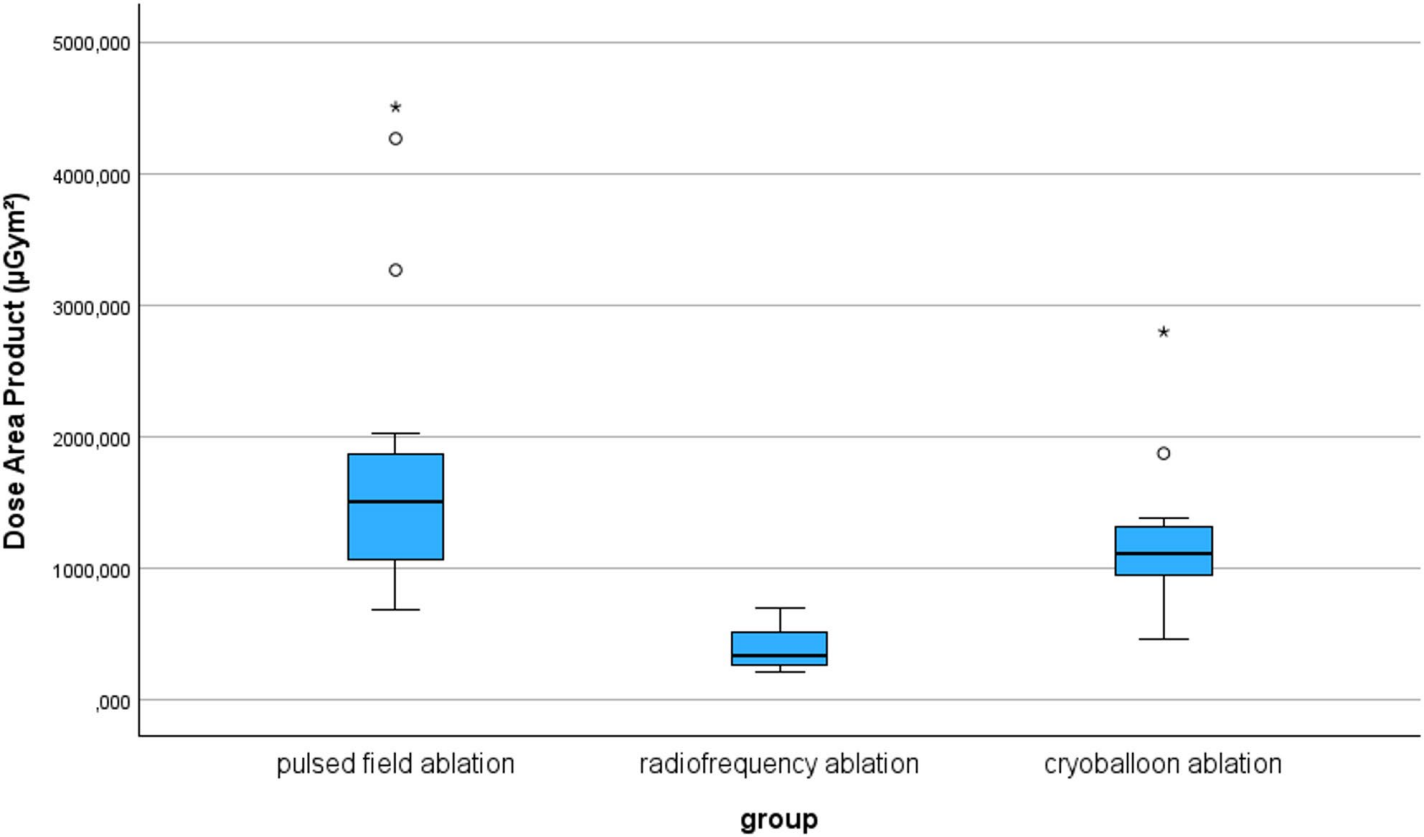
Dose Area Product in the respective ablation groups (*p* < 0.001), Legend: * outliers

Figure 1 shows the procedure duration and the catheter dwell time in the left atrium for each procedure. In the cryoballoon group, there is a relatively small interquartile range of 13 min in the ablation time, and 19 min in the catheter dwell time. However, there is also an extreme outlier in the boxplot with times over 2.5 times the interquartile range above the third quartile. This was a difficult procedure in which the isolation of the veins took an exceptionally long time. In comparison, the PFA group shows much greater scatter with the highest interquartile ranges. A comparison of the fluoroscopy times shows a clear difference between the groups. While the average fluoroscopy time for PFA was 35 min, it was lower in the cryoballoon group at 15 min and lowest in the HPSD-RF group with a value of 7 min. This difference was statistically significant (*p* < 0.001). In the pairwise comparison of the HPSD-RF group and the cryoballoon group, the difference was significant (*p* = 0.042); as was the comparison of PFA with the cryoballon group (*p* = 0.023). The differences in dose area product between the procedures were also significant. While the mean dose area product for PFA was 1507.10 µGym², it was lower for cryoballoon ablation with a value of 1115.80 µGym² and lowest for HPSD-RF with a value of 340.45 µGym². There was a statistically significant difference in the groups (*p* < 0.001). In the further pairwise analysis, there was a detectable difference between PFA and HPSD-RF (*p* < 0.001). The same was true for the comparison between HPSD-RF and cryoballoon ablation (*p* = 0.003). No significant difference was detected between PFA and cryoballoon ablation (*p* = 0.937). Figure 2 shows the different dose area products of the three procedures.

### Postoperative complications

Two postoperative complications were reported in the study. An inguinal arteriovenous-fistula requiring vascular surgery and prolonged hospitalization occurred in the PFA group. A pericardial tamponade occurred in the HPSD-RF group resulting in pericardiocentesis and a prolonged hospital stay. No complications were reported in the cryoballoon group. The frequency of a complication following the procedure was therefore 6.7% in the PFA group, 10.0% in the HPSD-RF group and 0.0% in the cryoballoon group. A statistical comparison of the three groups using the chi-square test did not reveal any detectable difference (*p*-value: 0.598). Further complications, such as injuries to the esophagus, the phrenic nerve or pulmonary vein stenosis were not observed. In addition, no strokes, transitory ischemic attacks (TIA) nor deaths occurred during the procedure.

### Atrial fibrillation recurrence rates

**Fig. 3:**
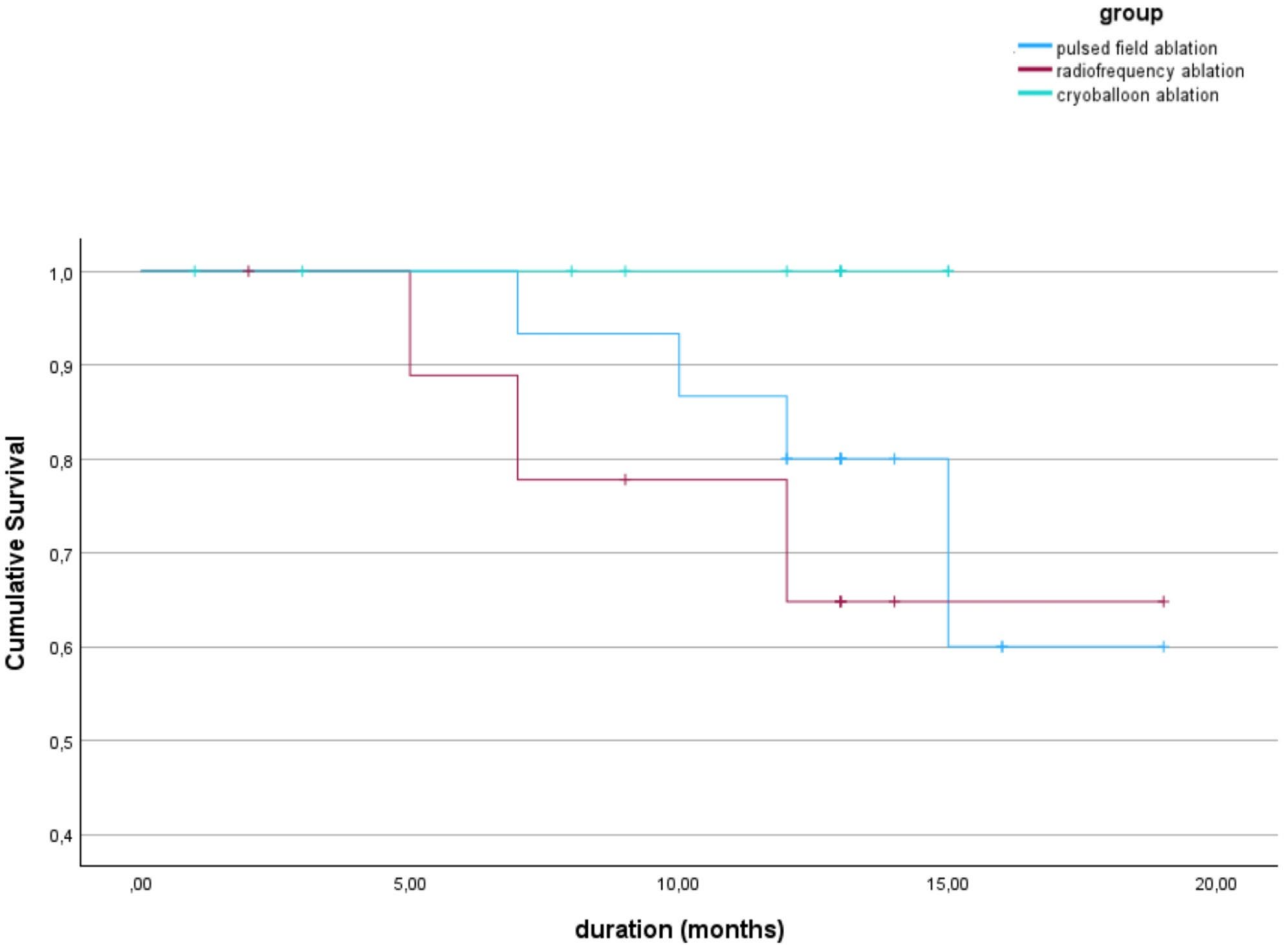
Kaplan-Meier curve showing recurrence rates in the respective ablation groups (*p* = 0.192)

The follow-up period was one year in all groups. Due to delays in some cases, the last follow-up appointment was delayed by a few months for some patients. However, since the last long-term ECG should not be neglected for the detection of recurrences, data from the delayed follow-up appointments were also taken into account. As already mentioned in the methods, the criterion of a recurrence was only considered fulfilled when the three-month blanking period was completed. A total of seven recurrences were observed during the follow-up period. These were detected either during the regular follow-up appointments, through emergency presentations of the participants in the clinic or, as an incidental finding in the control of pacemakers. Of these recurrences, four were observed in the PFA group (26.7%) and three in the HPSD-RF group (30.0%). In the third group of cryoballoon ablation, no recurrences were noted during the follow-up period. The recurrence rates are illustrated below by a Kaplan-Meier survival analysis in Fig. 3. As shown in the Kaplan-Meier curve, the earliest recurrence after ablation occurred in the HPSD-RF group after approximately 5 months. The last recurrence measured in the study was recorded after 15 months in the PFA group. At this time, cumulative recurrency- free was 100% in the cryoballoon ablation group, approx. 65% in the HPSD-RF group and 60% in the PFA group. The curve also shows that four participants in the cryoballoon group had already left post-treatment before the 10th month and therefore had to be censored. In HPSD-RF, two people had dropped out by this time. There were no drop-outs in the PFA group. No detectable difference between the groups was found in the log-rank test (*p*-value = 0.192).

### Quality of life

**Fig. 4:**
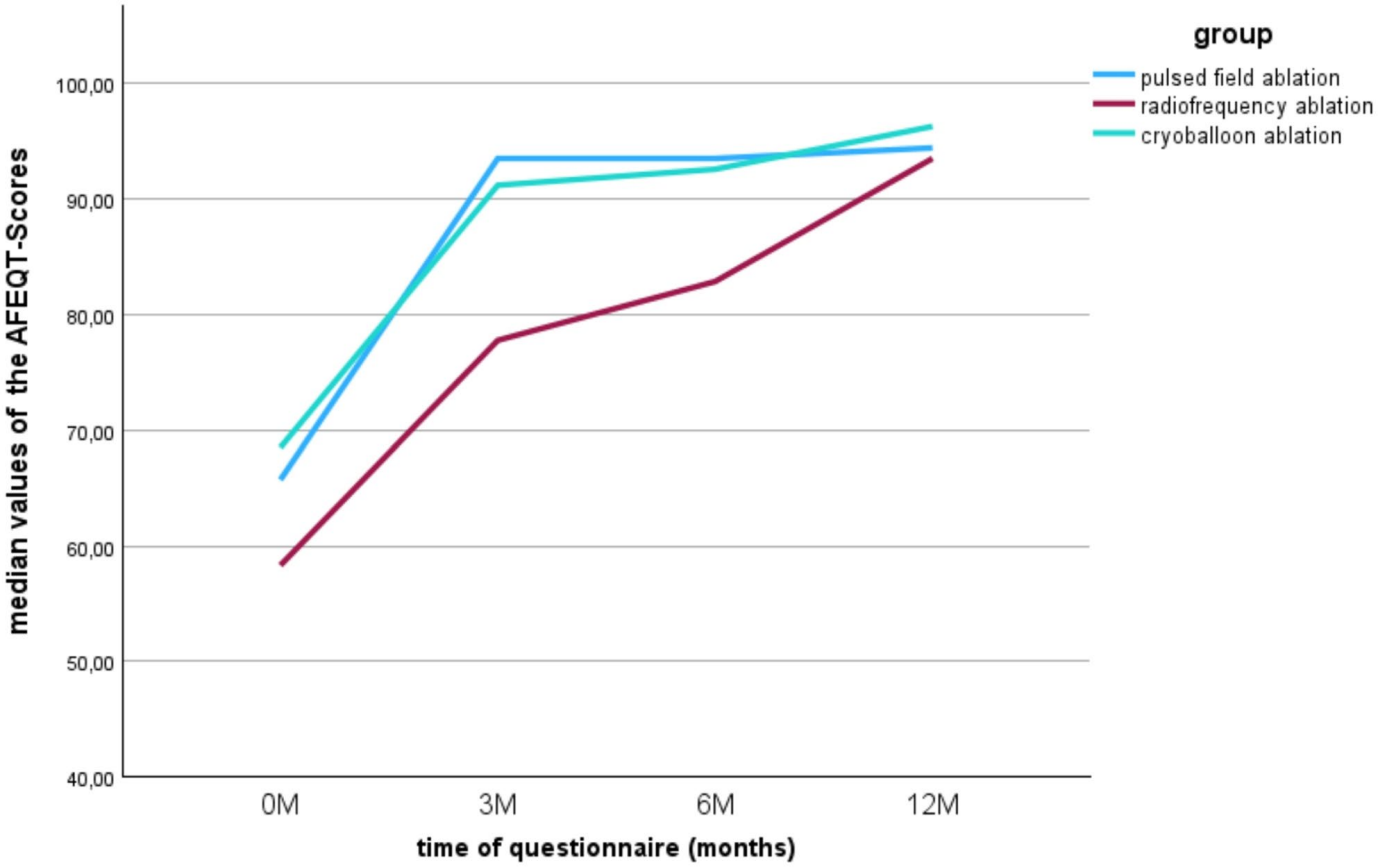
Increase in Quality of life (AFEQT-Scores) before and after ablation in the respective ablation groups (PFA: *p* = 0.04; HSPD: *p* = 0.022 und Cryoballoon: *p* = 0.015)

**Fig. 5:**
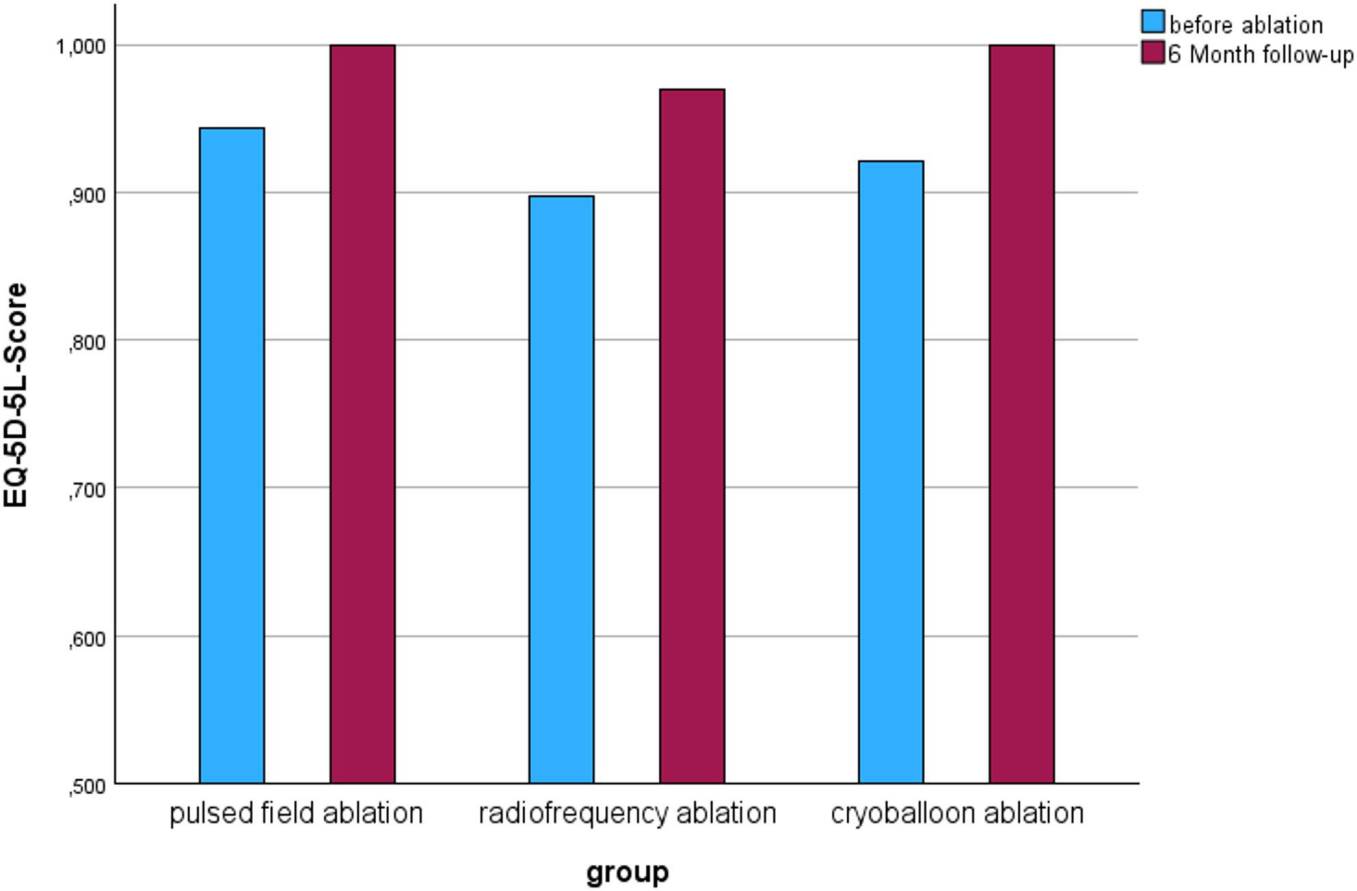
Quality of Life Graph (EQ-5D-5 L-Score) before ablation and at the 6 Month follow-up (*p* > 0.05)

Figure 4 illustrates the calculated scores from the AFEQT questionnaires at the various measurement times. The possible values between 0 and 100 can be categorized as follows: Values below 70 points correspond to a heavy symptom burden, values between 70 and 89 points correspond to a moderate symptom burden and values above 90 points correspond to a very low to no symptom burden due to atrial fibrillation disease [15]. All groups showed an initial high symptom burden of less than 70 points. The highest burden of symptoms due to atrial fibrillation was measured in the HPSD-RF group with a median of 58.34 points. In all three groups, higher values were achieved post-intervention and thus a lower described symptom burden. In the PFA group, the AFEQT score was a median of 93.52 points at the three- and six-month follow-up appointments and increased slightly to 94.44 points at the twelve-month appointment. In the HPSD-RF group, the median continued to rise at each examination time and reached a value of 93.52 points at the last appointment. The same development was observed in the cryoballoon group, in which the highest value of 96.30 points was achieved at the last follow-up appointment. The PFA group in particular showed a sharp increase in the AFEQT score between the first measurement date and the follow-up after three months, while the score hardly changed thereafter. The further statistical analysis included only the three- and six-month follow-up appointments due to an increased number of missing values at the twelve-month appointment. The Friedman test carried out indicated a demonstrable difference between the measurement times in the PFA group (*p* = 0.004). In the development of the AFEQT score of the HPSD-RF group, the slide shows a steady slope and thus a decreasing symptom burden over time. A statistical difference could also be shown (*p* = 0.022). In the cryoballoon group, the greatest increase in the score value occurred between the zero and three-month time points. Overall, a difference was shown for the measurements (*p* = 0.015). Figure 5 illustrates the increase in the EQ-5D-5 L score between the two measurement points. In all three groups, there is a clear increase in the value at the six-month point, and the highest possible value of 1 is reached in the PFA and cryoballoon groups. However, this increase in quality of life did not reach statistical significance.

## Discussion

The present study considers the ablation methods PFA, HPSD-RF ablation and cryoballoon ablation in paroxysmal atrial fibrillation. One point of consideration was the comparison of procedure data, which included procedure duration, catheter dwell-times in the left atrium and fluoroscopy parameters. Procedure duration and the catheter dwell-time were shortest for cryoballoon ablation, followed by PFA. The longest procedures were noted for HPSD-RF. We performed HPSD-RF as opposed to LPLD-RF in the hope of keeping procedure times down and comparative with PFA and cryoballoon ablation. In the CIRCA-Dose study, the procedure times for radiofrequency ablation were also longer than those for cryoballoon ablation [16]. The procedure times in the aforementioned study were higher for both cryoballoon ablation and radiofrequency ablation than in the study presented here. A possible explanation for this is a 20-minute observation time after the ablation lesions in the CIRCA-DOSE study [16]. This distribution of procedure times between the two methods was also shown in the FIRE AND ICE study, which demonstrated the non-inferiority of the cryoballoon in 2016 [10]. PFA was in-between the two other procedures in terms of procedure times and showed shorter times than in comparative studies such as the PULSED AF pivotal study or the ADVENT study [17, 18]. In these studies, an observation time of 20 min were also included in the ablations performed [17, 18]. The different duration of the procedures is mainly due to the different techniques. Both cryoballoon ablation and pulsed-field ablation are single-shot procedures, although rotation of the PFA-Device is required, while radiofrequency ablation works with point-to-point lesions.

Further intraprocedural differences were observed for the fluoroscopy times and dose-area product. RF showed significantly shorter fluoroscopy times than cryoballoon ablation, which was also demonstrated in both the CIRCA-DOSE study and the FIRE AND ICE study [10, 16]. This lower fluoroscopy time in the HPSD-RF group is interesting and corresponds to the expected results, as the anatomical orientation during radiofrequency ablation was supported and certainly influenced by 3D-mapping. Electroanatomical mapping was also used for pulsed-field ablation. However, the fluoroscopy time was highest in pulsed-field ablation despite mapping, which could be explained by a longer procedure duration than in cryoballoon ablation, despite both being singe-shot ablation procedures. Compared to the PULSED-AF pivotal study, the fluoroscopy time was higher in this study [17]. Pulsed-field ablation is the newest of the three ablation procedures and, as experience with this new procedure is gathered, shorter procedure times and fluoroscopy times are to be expected.

In addition to the procedural data, the presence of complications plays an important role in determining the safety profile of the individual ablation methods. Only two complications were observed in the study. One pericardial tamponade was observed in the HPSD-RF group, possibly related to the transeptal access, and one inguinal arteriovenous fistula in the PFA group which may have been due to larger sheaths required for vascular access in PFA. In the ADVENT study, in which PFA was investigated, one serious complication occurred during vascular access in 305 procedures [18]. Vascular complications are therefore rare. Atrioesophageal fistulas, pulmonary vein stenoses, strokes or other complications including deaths were not seen in the study.

The success of the ablation procedures with regard to recurrence rates are the central question of this study. The first human studies on PFA by Reddy et al. showed an average freedom from atrial arrhythmias of approx. 78.5% after a one-year follow-up period [19]. All participants included in the study had symptomatic and paroxysmal atrial fibrillation [19]. In the PULSED-AF study, the efficacy of the procedure was compared in paroxysmal and persistent atrial fibrillation. After one year, an efficacy of approx. 66% was shown for paroxysmal and approx. 55% for persistent atrial fibrillation [17]. A comparison of the recurrence rates with these studies is difficult, as stricter post-interventional monitoring, such as weekly reviews in some cases, were carried out in these larger studies. Nevertheless, it can be said that no recurrence was detected in 73.3% of the participants in this study who were treated using PFA. This means that the results are in line with the success rates observed to date for paroxysmal atrial fibrillation. A similar success rate of 70% was observed in the HPSD-RF group. This efficacy is comparable to the 73% success of RF in the study by Ali et al. [20]. In the FIRE AND ICE study, freedom from recurrence was slightly lower after one year at 64% [10]. No recurrences were detected in the cryoballoon ablation group, although this should be interpreted cautiously due to small sample size and early censoring. In the literature, the success rate of cryoballoon ablation was similar to other ablation methods. In the FIRE AND ICE study, it was 65% after one year [10], while in comparable studies it was between 73 and 75% [20, 21]. Thus, the group of participants in this study who were treated with a cryoballoon showed better results than expected. It should be borne in mind that some participants in this group left the observation earlier than expected and participants in the other two methods were sometimes examined for longer. In addition, the log-rank test of the Kaplan-Meier curve showed no statistical difference between the groups (*p*-value = 0.192).

According to the ESC guidelines for atrial fibrillation from 2020, catheter ablation serves in particular to improve the quality of life of symptomatic patients [1]. The extent to which this goal is achieved by the various ablation procedures is therefore an important question in this study. The AFEQT questionnaire showed a clear post-interventional reduction in symptom burden. This improvement was statistically proven in all groups. Thus, the primary goal of symptom reduction in the ESC guidelines was achieved by all three tested procedures. In all three groups, it was also shown that the median continued to increase until the last follow-up appointment. This raises the question of whether this trend would have continued over a longer observation period. In the MANTRA-PAF study, a longer-term assessment of the burden of atrial fibrillation after ablation with radiofrequency ablation was carried out. It was shown that the quality of life continued to increase steadily up to the 24-month follow-up appointment [22]. Thus, there are indications that the symptom burden continues to decrease or at least remains stable in the long term. The reduction in symptom burden was also demonstrated in other studies. For example, participants in the PUSLED-AF study showed an average improvement in the AFEQT score of 25.9 points after PFA [23]. Whilst the AFEQT questionnaire is specific for atrial fibrillation and provides an overall score for symptoms, daily activities, treatment concerns and treatment satisfaction, the EQ-5D-5 L questionnaire is a generic, descriptive system which measures the general functional limitation of the respondents in everyday life, as well as subjective health in the form of an analog scale. No demonstrable improvement could be detected in the groups for the EQ-5D-5 L determined for the everyday dimensions. Overall, the initial values were already quite high, with a score of at least 0.898. In the study by Gupta et al., subjective health was measured using the EQ-5D-5 L before and after radiofrequency ablation. This showed comparable changes with an increase in the mean score from 72.7 to 81.4 points [24]. An interesting aspect with regard to the questionnaires is the correlation between the symptom burden determined in the AFEQT score and the general health impairment in the EQ-5D-5 L. While the symptom burden in the AFEQT score showed a clear improvement, only moderate changes were noticeable in the EQ-5D-5 L. It should also be noted however, that a subjective health assessment is multifactorial and could be influenced by other factors.

### Study limitations

In the present study, important findings were obtained with regard to the effectiveness of various ablations in atrial fibrillation. Nevertheless, some limitations of the study must be taken into account and the method must be viewed critically. First and foremost, we acknowledge that the groups had only a small patient population, which limits the significance of the study and underpowered to detect meaningful differences in recurrence rates. An important cause of this limitation was the Covid-19 pandemic, which occurred at the same time as the study was being conducted. Both the recruitment of patients and the implementation of the interventions were made considerably more difficult. This restriction due to the pandemic resulted in a much lower number of electrophysiological interventions worldwide [25]. The study by Pius et al. examined precisely these effects for patients with atrial fibrillation who were awaiting ablation. It was found that symptomatic patients often did not seek medical attention during the pandemic, whereas they would have done so under normal circumstances [26]. It can therefore be assumed that without these pandemic conditions, a faster and larger number of recruitments could have been made possible. This has made the study more exploratory and hypothesis-generating. Secondly, patients were non-randomly assigned to ablation strategies, so that selection bias cannot be excluded. However, as the basic clinical data of the groups proved to be sufficiently similar statistically, a largely homogeneous distribution of participants could be assumed. Also worth mentioning is the fact that the present study is a monocentric study in which all interventions were performed in the same hospital and thus the framework conditions of the interventions could be designed uniformly for the most part. Thirdly, PFA is a new procedure and operator preference as well as learning-curve effects may have influenced outcomes. Fourthly, another limitation of the study was individual missing data from certain participants. Some participants discontinued aftercare for personal reasons. Frequent reasons for this were other health problems, professional or personal commitments or in some cases long journeys to the university hospital through the region of Mecklenburg-Vorpommern. Due to the installation and performance of the long-term ECGs, it was necessary for the participants to present themselves twice per follow-up appointment and to organize a referral from a cardiologist in private practice. Although some long-term examinations could be carried out with the help of general practitioners or cardiology colleagues, the effort involved in traveling was often too high. The delays in follow-up care led to follow-up periods of varying lengths, which limited the direct comparability of the groups. However, this also resulted in longer observation periods than twelve months, which gave initial indications of even longer-term success rates for the participants. Fifthly, despite the follow-up measures, no permanent monitoring of the heart rhythm could be realized. Particularly in the case of asymptomatic recurrences, it must be questioned whether these patients could be recognized with sufficient certainty. For future studies, closer monitoring, for example with telemetric transmission or the use of event recorders, would be an option for more permanent rhythm monitoring.

## Conclusion

In the present study, the ablation methods of PFA, HPSD-RF and cryoballoon ablation were investigated and a comparable reduction in symptom burden could be achieved in all three ablation procedures with no statistically significant differences with respect to atrial fibrillation recurrence. The present study showed important findings for the comparison of the ablation methods in a small number of participants and was thus able to provide indicative results for the effectiveness of the procedures. For a more comprehensive and precise analysis of PFA, HPSD-RF and cryoballoon ablation, larger studies are needed to compare the success rates.

## Data Availability

The datasets used and/or analysed during the current study are available from the corresponding author on reasonable request.

## Abbreviations

RF: Radiofrequency
HPSD-RF: High-Power, Short-Duration Radiofrequency
LPLD-RF: Low-Power, Long-Duration Radiofrequency
PFA: Pulsed-Field Ablation
LA: Left Atrium
LV-EF: Left Ventricular Ejection Fraction
TEE: Transesophageal Echocardiography
ECG: Electrocardiogram
AFEQT: Atrial Fibrillation Effect on Quality of Life
EQ-5D-5L: EuroQol-5 Dimensions-5 Levels
PCI: Percutaneous Coronary Intervention
NYHA: New York Heart Association
DOAC: Direct Oral Anticoagulant
